# Solar-Cycle Variability Results from the *Solar Radiation and Climate Experiment* (SORCE) Mission

**DOI:** 10.1007/s11207-022-01980-z

**Published:** 2022-04-06

**Authors:** Thomas N. Woods, Jerald W. Harder, Greg Kopp, Martin Snow

**Affiliations:** 1 Laboratory for Atmospheric and Space Physics, University of Colorado, 3665 Discovery Dr., Boulder, CO 80303 USA; 2 South African National Space Agency Hospital Street, Hermanus, 7200 South Africa; 3 Department of Physics and Astronomy Robert Sobukwe Road, University of the Western Cape, Bellville, 7535 South Africa

**Keywords:** Total solar irradiance, Solar spectral irradiance, Solar-cycle variability, Sun–climate observations

## Abstract

The *Solar Radiation and Climate Experiment* (SORCE) was a NASA mission that operated from 2003 to 2020 to provide key climate-monitoring measurements of total solar irradiance (TSI) and solar spectral irradiance (SSI). This 17-year mission made TSI and SSI observations during the declining phase of Solar Cycle 23, during all of Solar Cycle 24, and at the very beginning of Solar Cycle 25. The SORCE solar-variability results include comparisons of the solar irradiance observed during Solar Cycles 23 and 24 and the solar-cycle minima levels in 2008 – 2009 and 2019 – 2020. The differences between these two minima are very small and are not significantly above the estimate of instrument stability over the 11-year period. There are differences in the SSI variability for Solar Cycles 23 and 24, notably for wavelengths longer than 250 nm. Consistency comparisons with SORCE variability on solar-rotation timescales and solar-irradiance model predictions suggest that the SORCE Solar Cycle 24 SSI results might be more accurate than the SORCE Solar Cycle 23 results. The SORCE solar-variability results have been useful for many Sun–climate studies and will continue to serve as a reference for comparisons with future missions studying solar variability.

## Introduction

The *Solar Radiation and Climate Experiment* (SORCE) provided crucial solar-irradiance observations during its 17 years of operations for NASA’s Earth Science program (e.g. Woods et al., 2021). Solar irradiance is the principal energy input to the global climate system and is critical for studying the radiative-energy balance (e.g. Wild et al., 2015; L’Ecuyer et al., 2015), atmosphere photochemistry (e.g. Haigh et al., 2010; Ermolli et al., 2013), and solar influence on global and regional climate change (e.g. Lean and Rind, 2008; Scaife et al., 2013). The SORCE observations include the total (TSI) and the solar spectral irradiance (SSI), both being important for NOAA’s Climate Data Record (CDR) program (e.g. Coddington et al., 2016). A key motivation for extending the SORCE measurements well past its five-year prime mission was to prevent a gap in the TSI and SSI climate records (Woods et al., 2021). With the launch of NASA’s *Total and Spectral Solar Irradiance Sensor* (TSIS-1) to the *International Space Station* (ISS) in December 2017 and TSIS-1 routine solar observations beginning in March 2018, there was a 22-month overlap of SORCE and TSIS-1 observations before the SORCE mission was terminated. The SORCE 17-year mission is notable for its TSI and SSI observations during the descending phase of Solar Cycle 23 from 2003 to 2008, through an unusually extended minimum at the end of Cycle 23 in 2008 – 2009 (e.g. Russell, Luhmann, and Jian, 2010), and over the full period of Solar Cycle 24 (2009 – 2020), which is the least active cycle in 100 years (e.g. Hathaway and Upton, 2014). As presented in Section 2, the comparison of SORCE’s solar-irradiance measurements near cycle maxima to cycle minima provides results about the variability of the solar 11-year activity cycle, which is manifested by the solar 22-year magnetic cycle. As there are differences in the SORCE variability results for Solar Cycles 23 and 24, the irradiance variability for solar 27-day rotation periods is also examined for a period in 2012 when TSI and ultraviolet (UV) irradiance were in-phase with each other (Section 3). The SORCE mission is also remarkable in observing two solar-cycle minima periods with the same instruments, so estimates/constraints of the solar-irradiance secular trends are possible (Section 4). The solar-cycle variability results from SORCE are also compared to irradiance models and SSI composites to highlight progress in understanding solar-cycle variability. The key conclusions and a few notes where future studies are required are provided in Section 5.

Although comparison of irradiance variability on the solar-cycle timescale from TSIS-1 is not feasible yet, we do mention some comparisons of SORCE and TSIS-1 observations for radiometric validation. Harder et al. (2022) present the revised SORCE/SIM data product that adopts the SSI irradiance level from more accurate TSIS-1/SIM observations between March 2018 and February 2020. Similarly, the SSI3 composite by Woods and DeLand (2021) adopts the TSIS-1/SIM reference spectrum in March 2018 (Richard et al., 2020) as the basis for scaling the SORCE/SIM observations for the SSI3 composite. In both cases, the adjustments for the SORCE/SIM SSI data are up to 5 % decrease for wavelengths longer than 900 nm. The revised SORCE/SIM product, called the TSIS1 SIM Adjusted Values (TAV V02: Harder et al., 2022), also has adjustments up to 5 % in the 270 – 340 nm UV range. The SSI3 Composite only uses the SORCE/SIM data above 500 nm, so it does not include those adjustments in the UV range. Kopp (2021) provides a comparison of SORCE/TIM to all of the overlapping TSI observations, including those from TSIS-1/TIM. This TSI comparison shows good agreement for all of the TIM instruments. In addition, Mauceri et al. (2020b) provide detailed comparisons of SIM SSI and TIM TSI measurements from SORCE and TSIS-1. Another noteworthy comparison is that the *Compact-SIM* (CSIM) cubesat observations agree with the TSIS-1/SIM SSI observations to within 0.5 % (Richard et al., 2019).

A quick introduction to the SORCE instruments is provided here. SORCE’s *Total Irradiance Monitor* (TIM: Kopp and Lawrence, 2005; Kopp, Heuerman, and Lawrence, 2005) is an ambient-temperature electrical substitution radiometer (ESR) with four channels, with one channel used for daily TSI observations and the other channels used less frequently to track degradation of the primary channel due to solar exposure. The *Spectral Irradiance Monitor* (SIM: Harder et al., 2005a,b) (240 – 2413 nm), *SOLar STellar Irradiance Comparison Experiment* (SOLSTICE: McClintock, Rottman, and Woods, 2005; McClintock, Snow, and Woods, 2005) (115 – 308 nm), and *X-ray UV Photometer System* (XPS: Woods and Rottman, 2005; Woods, Rottman, and Vest, 2005) (0.1 – 40 nm and H i 121.6 nm) measure the spectral composition of the total irradiance. The SORCE/SIM is a dual-channel prism spectrometer with a spectral resolution of 1 – 30 nm, and SIM measures the SSI in the near-ultraviolet (NUV: 300 – 400 nm), visible (VIS: 400 – 800 nm), and near-infrared (NIR: 800 – 2000 nm) ranges. One SIM channel is used for the daily SSI observations, and the other channel is used about once a month to track instrument degradation. The SORCE/SOLSTICE is a set of two grating spectrometers with a spectral resolution of 0.1 nm, and SOLSTICE measures the SSI in the far-ultraviolet (FUV: 120 – 200 nm) and mid-ultraviolet (MUV: 200 – 300 nm) ranges. Both SOLSTICE-A and SOLSTICE-B are used daily for solar observations, and stellar observations of stable main-sequence O–B stars are used to track the degradation of the SOLSTICE channels. The SORCE/XPS is a set of photometers with ≈ 10 nm bandpass filters covering the extreme ultraviolet (EUV: 10 – 120 nm) and soft X-ray (SXR: 0.1 – 10 nm) ranges. The XPS Level 4 spectral model in 1-nm bins for the 1 – 27 nm range (Woods and Elliott, 2022) is used for this variability study instead of the XPS Level 3 broadband irradiance products. To fill the spectral gap in the SORCE SSI measurements between 27 nm and 115 nm, we use the solar EUV irradiance measurements from the *Solar EUV Experiment* (SEE) onboard NASA’s *Thermosphere Ionosphere Mesosphere Energetics and Dynamics* (TIMED) mission (Woods et al., 2005).

This article focuses on the solar-variability results from the SORCE mission on solar-cycle timescales, which we also refer to as the solar-cycle variability. The final SORCE archived data products are used, and they are TIM Level 3 Version 19, SIM Level 3 Version 27, SOLSTICE Level 3 Version 18, and XPS Level 4 Version 12. The TIMED/SEE Level 3 Version 12 data are also used for this analysis. The uncertainty/stability estimates for these instruments are shown in Table 1. Instrument-degradation trending is critical for the accuracy of solar-variability estimates, and those trends are presented in companion articles about the SORCE instruments and their degradation trends as part of their final science data-processing algorithms (TIM: Kopp, 2021; SIM: Harder et al., 2022; SOLSTICE: Snow et al., 2022; XPS: Woods and Elliott, 2022). There is also another SORCE companion article that introduces some of the Sun–climate topics and an overview of instrument and spacecraft performance over its 17-year mission (Woods et al., 2021).

**Table 1:** Instrument accuracy and long-term repeatability estimates. Accuracy is the NIST-defined combined standard uncertainty (Taylor and Kuyatt, 1994) at the beginning of the mission, and long-term repeatability (stability) is the measurement precision on multi-year timescales.

Instrument	Measurement	Spectral range/resolution	Accuracy	Long-term repeatability
SORCE/TIM	TSI 4 cones	N/A	0.035 %	0.001 % year^−1^
SORCE/SIM	SSI 2 spectrometers	200 – 2400 nm/2 – 30 nm	2 %	0.01 – 0.03 % year^−1^ *λ* dependent
SORCE/SOLSTICE	SSI 2 spectrometers	115 – 308 nm/0.1 nm	1 – 3 % *λ* dependent	0.2 – 0.5 % year^−1^ *λ* dependent
SORCE/XPS	SSI 8 photometers	0.1 – 27 & 121 nm/7 – 10 nm	12 – 24 % *λ* dependent	1.5 % year
TIMED/EGS	SSI 1 spectrograph	27 – 190 nm/0.4 nm	10 – 20 % *λ* dependent	1.5 % year^−1^

The focus here is on the 11-year solar-activity cycle, which is a consequence of the 22-year solar magnetic cycle (e.g. Cliver, 2014). We discuss in detail the significant SSI-variability differences between the observed and modeled spectra. As detailed by Ermolli et al. (2013), this spectrally dependent variability is critical for understanding the solar forcings and their impacts in Earth’s climate system at inter-annual and decadal timescales. To assist in addressing those concerns, models of the SSI variability are being refined based on the SORCE observations. Ermolli et al. (2013) provide a comparison of model estimates of solar-cycle variability for the NUV-VIS-NIR and show significant differences among the model-variability estimates and the early SORCE/SIM results about SSI variability as presented by Harder et al. (2009). The Ermolli et al. (2013) primary concern is that the large NUV in-phase variability and large visible out-of-phase variability from Harder et al. (2009) are not reproduced in the NRLSSI model (Lean et al., 2005) and SATIRE model (Krivova, Solanki, and Unruh, 2011) but semi-empirical models such as SRPM (Fontenla et al., 2011) and COSI (Shapiro et al., 2010) produce out-of-phase cycle trends in the visible and infrared portions of the spectrum.

Since the Ermolli et al. (2013) comparisons, Ball et al. (2014) have updated the SATIRE model, and Coddington et al. (2016) and Lean et al. (2020) have updated the NRLSSI model. Additionally, the degradation trends for the SORCE instruments have been refined, bringing measurements and models into better agreement. While the differences between observations and models have decreased, notably in the NUV and visible regions, there remain differences in the phasing of the solar-cycle variability in the NIR region (Woods and DeLand, 2021). The new SSI measurements with improved accuracy and stability by TSIS-1/SIM are anticipated to resolve some of the remaining differences. However, the low solar activity so far during the TSIS-1 mission (2018 to present) does not provide enough variability over the current solar cycle to accurately address those differences yet. For example, the most magnetically active solar rotation during the TSIS-1 mission, so far, is four times smaller than the typical solar rotation observed during the SORCE mission.

**Figure 1:**
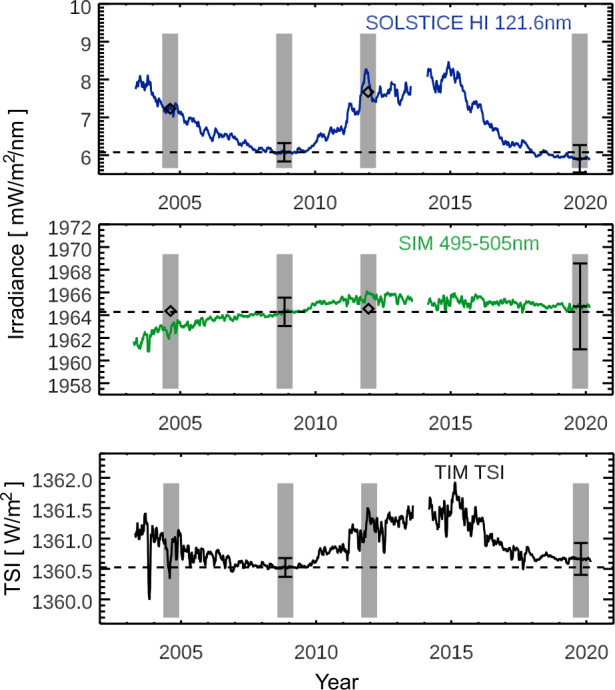
The SORCE observations span 17 years, covering the declining phase of Solar Cycle 23, cycle minimum in 2008 – 2009, all of Solar Cycle 24, and the next cycle minimum in 2019. The select wavelengths are examples for emissions from the photosphere (500 nm) and the chromosphere/transition region (121.6 nm). The variability for the TSI and visible (and NIR) is dominated by dark sunspots and bright faculae, and the UV variability has only bright contributions from active regions and active networks. The *gray* regions indicate the 217-day periods (eight solar rotations) for solar-cycle maxima and minima as used for solar-cycle variability results shown in Figures 3 and 4. The *diamonds* are the SSI3 composite values for those maximum periods (Woods and DeLand, 2021). The error bars at cycle minima show the uncertainty for these measurements. These time series include five-day smoothing of the SORCE data.

We will present the solar-cycle variability for the final, archived SORCE data products, but the SSI variability in some wavelength regions remains controversial and unresolved. One such example is initially discussed for the 500-nm time series with the SORCE/SIM data, as shown in Figure 1. Wehrli, Schmutz, and Shapiro (2013) and Haberreiter et al. (2017) point out that there are inconsistencies in the SORCE/SIM variability between Solar Cycles 23 and 24 for the irradiance near 500 nm. As shown in Figure 1, the 500-nm irradiance time series shows a rise of irradiance during the decline of Solar Cycle 23 (2003 – 2009) and a slight irradiance increase during the Solar Cycle 24 rise (2009 – 2013). It seems unlikely that the irradiance would behave significantly differently during two different solar cycles. We note, however, that those trends are within the SIM stability estimate of ≈ 0.1 % over a ten-year period. The SORCE/SIM was a first-generation instrument and had stability uncertainties comparable to solar-cycle variability levels. Thus, the SIM sensors were improved for its second generation on TSIS-1 (Richard et al., 2020) and its third generation on *Compact-SIM* (CSIM CubeSat; Richard et al., 2019). We anticipate that these newer versions of SIM could resolve most of the current SSI solar-cycle variability controversy once activity ramps up to Cycle 25 maximum during the TSIS-1 and CSIM missions. In addition to showing here the solar-cycle variability for the currently archived SORCE data products, we will show additional variability results with the SORCE data for solar-rotation periods as one approach to identify similarities in SSI variability between measurements and models.

## Irradiance Variability for Solar-Cycle Timescales

**Figure 2:**
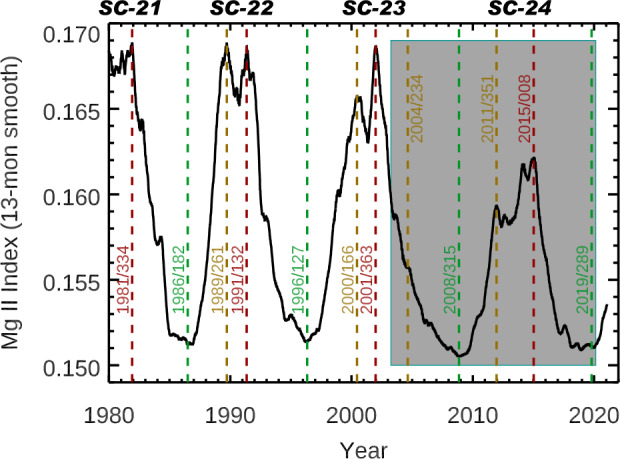
The Mg ii index smoothed over 217 days is used to identify solar-cycle minima (*green*) and maxima (*red and gold*) dates [year/day of year format]. There are sometimes two or more peaks during cycle maxima. The *gray region* identifies times during the SORCE mission. Because the SORCE mission did not begin until after the Solar Cycle 23 (SC23) maxima, a special period in 2004 is identified for discussing SC23 variability during the SORCE mission.

The Sun varies on all timescales: from seconds to minutes for solar flares; from hours to days for solar-active regions (e.g. sunspots) emergence and evolution; for the approximate 27-day rotation of the Sun; over many years for the approximate 11-year solar-activity cycle; and over many decades and centuries for secular changes likely related to the solar-dynamo evolution. To identify the solar-cycle minimum and maximum dates, the Bremen Mg-index was smoothed over 217 days (approximately 8 solar rotations, or 7 months). As a 217-day period is a significant part of the solar cycle, other solar proxies, also smoothed by 217 days, provide similar dates for the cycle minima and maxima. As illustrated in Figure 2 for the past four solar cycles, the solar-activity cycles do not have a fixed 11-year period. This analysis shows a 9.7-year period for Solar Cycle 22 (SC22: 1986 – 1996), a 12.8-year period for SC23 (1996 – 2009), and a 10.6-year period for SC24 (2009 – 2019). As noted in Figure 2 and Table 2, it is typical for the recent solar cycles to have two maxima, which is partially caused by the northern-hemisphere active regions peaking at a different time from those in the southern hemisphere (e.g. Veronig et al., 2021). Furthermore, different wavelengths can have different dates for their cycle minima and maxima, but generally the dates are within a few months of each other when using smoothed data over many months. Table 2 lists the dates for the cycle minima and maxima during Solar Cycles 22 – 24 for the 217-day smoothed Mg ii index. The SORCE mission started after the Solar Cycle 23 maxima, so an alternative date in 2004 is selected early in the SORCE mission that corresponds to a peak in the 81-day smoothed Mg ii index and after the SIM prism-scan operations changed in April 2004. This selected 2004 period early in the SORCE mission is just moderate solar activity during the Cycle 23 declining phase, but we consider it appropriate for studying Solar Cycle 23 variability for the SORCE mission. As discussed more by Woods et al. (2021), there were some SORCE battery-capacity issues that became more serious in 2013. Therefore, the first maximum in Solar Cycle 24 is chosen as the preferred maximum for the SORCE analysis. As shown in Figure 2 for the Mg ii index, the SORCE chosen date of 2004/234, that is day of year (DOY) 234 of 2004, for Solar Cycle 23 is less active than the Solar Cycle 24 maximum date of 2011/351.

**Table 2:** Solar-cycle minima and maxima dates. These dates are based on 217-day smoothing of the Bremen Mg ii index. The Solar Cycle 23 maxima do not occur during the SORCE mission, so an alternative date is chosen that is a peak for 81-day smoothing of the Mg index; this 2004 date represents moderate activity during Cycle 23 decline.

Solar-cycle number	Start minimum [Year/DOY]	Maximum 1 Maximum 2 [Year/DOY]	“Maximum” for SORCE mission [Year/DOY]	End minimum [Year/DOY]
22	1986/182	1989/261 1991/132	N/A	1996/127
23	1996/127	2000/166 2001/363	2004/234	2008/315
24	2008/315	2011/351 2015/008	2011/351	2019/289

The solar-cycle variability in irradiance units is calculated as the maximum irradiance minus the minimum irradiance. We also present unitless relative variability that is calculated as the solar-cycle variability in irradiance units divided by the solar-minimum irradiance. For these analyses, the 217-day averages centered on the minimum and maximum dates of each cycle are used for the variability calculation. For comparison of the Solar Cycles 23 and 24 variability results from the SORCE mission, the solar-minimum irradiance is the 217-day average centered on day 2008/315. We note that the 2008 – 2009 minimum is an important period that has been extensively studied for the Whole Heliosphere Interval (WHI) international campaign (e.g. Woods et al., 2009; Gibson et al., 2011; Thompson et al., 2011).

**Figure 3:**
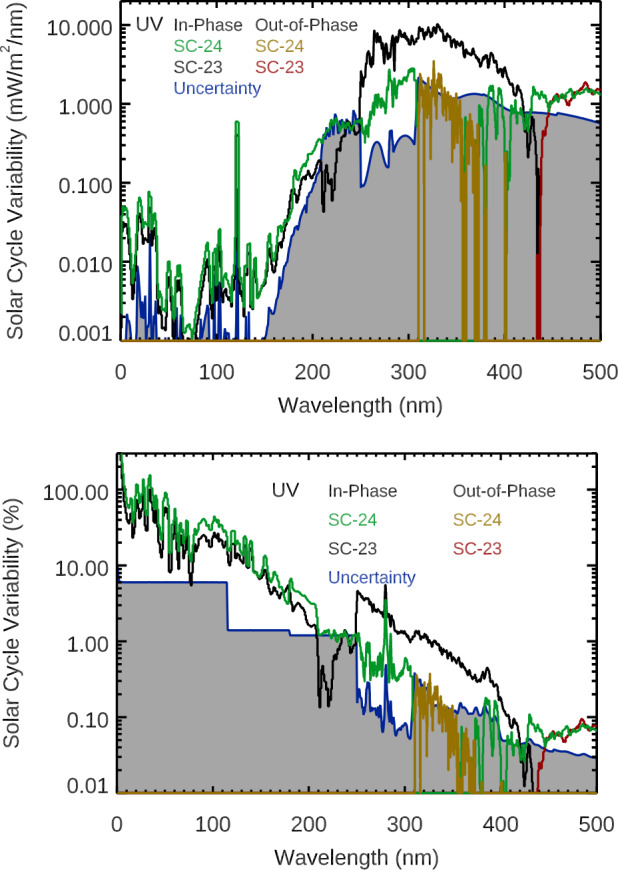
The spectral dependences of solar-cycle variability results for the SORCE data in the EUV-UV-NUV ranges are shown for Solar Cycle 23 (*black and red*, 2004 period minus 2008 – 2009 period) and Solar Cycle 24 (*green and gold*, 2011 – 2012 period minus 2008 – 2009 period). The *top panel* shows variability in irradiance units (maximum–minimum), and the *bottom panel* shows relative variability in percentage change from the 2008 – 2009 minimum level. The SORCE/SIM data are used for wavelengths longer than 250 nm, and the UV data at wavelengths shorter than 250 nm are from SORCE/SOLSTICE, TIMED/SEE, and SORCE/XPS. *The two colors for each solar cycle* represent the in-phase (positive) variability and the out-of-phase (negative) variability. The variability uncertainties are shown as the *blue line* and *gray-shaded regions*.

**Figure 4:**
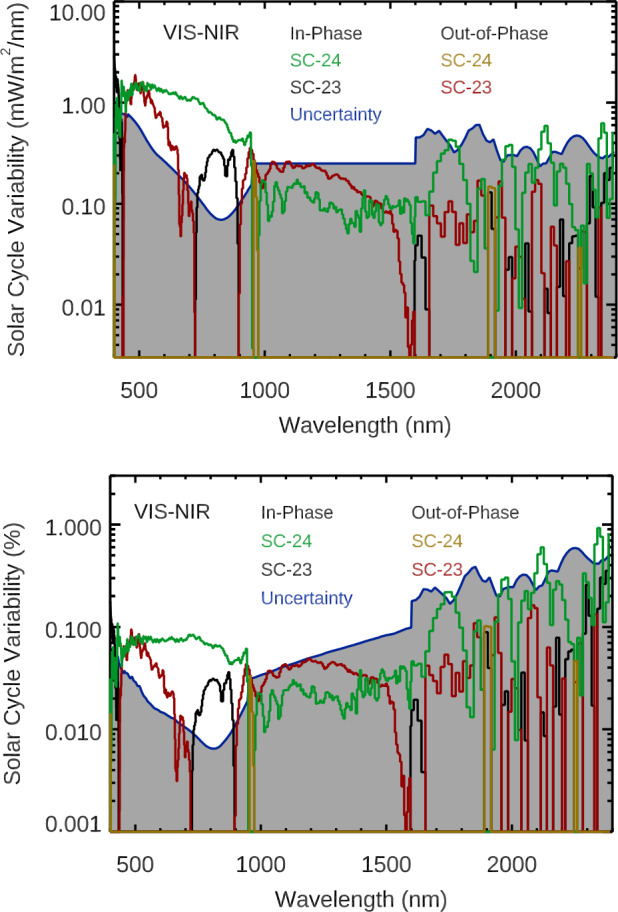
The spectral dependences of solar-cycle variability results for the SORCE data in the VIS-NIR ranges are shown for Solar Cycle 23 (*black and red*, 2004 period minus 2008 – 2009 period) and Solar Cycle 24 (*green and gold*, 2011 – 2012 period minus 2008 – 2009 period). The *top panel* shows variability in irradiance units (maximum–minimum), and the *bottom panel* shows relative variability in percentage change from the 2008 – 2009 minimum level. The SORCE/SIM data are used for wavelengths longer than 250 nm. *The two colors for each solar cycle* represent the in-phase (positive) variability and the out-of-phase (negative) variability. The variability uncertainties are shown as the *blue line* and *gray-shaded regions*. Note that the uncertainties in many wavelengths in the NIR range exceed the measured variability.

The solar-cycle variability results with the SORCE data are shown in Figures 3 and 4 for Solar Cycles 23 and 24. The TSI solar-cycle maxima from the SORCE/TIM are 1360.83 W m^−2^ and 1361.22 W m^−2^ for the SORCE-selected maximum dates in Solar Cycles 23 and 24, respectively. With the SORCE/TSI minimum value of 1360.52 W m^−2^, these TSI relative variability values are 0.023 % and 0.051 %. The largest amount of SSI variability in irradiance units is for wavelengths between 300 nm and 700 nm, so those wavelengths can have larger influences in Earth’s atmosphere and at the surface for Sun–climate effects. From examining the relative variability results (unitless), the EUV/SXR wavelengths vary the most by 30 % or more, followed by 3 – 25 % variability for the FUV wavelengths. These highly variable emissions are from the hot corona and transition-region emissions. The amount of relative variability reduces to less than a percent for the NUV-VIS-NIR ranges, approaching about 0.05 %, which is comparable to TSI relative variability. However, there are many wavelengths in the VIS-NIR ranges that show out-of-phase (negative) variability, especially so for Solar Cycle 23 from the SORCE/SIM data.

The solar-cycle variability uncertainty [$\sigma $_var_] is primarily from the estimate of instrument long-term repeatability (stability) between the minimum and maximum periods. As shown in Equation 1, the variability uncertainty is calculated as the root sum square (RSS) of the maximum irradiance precision [$p$_max_], the minimum irradiance precision [$p$_min_], and the stability estimate between minimum and maximum [$S$($t$_min_, $t$_max_)]:

1 $$ \sigma _{\operatorname{var}} = \sqrt{\left ( p_{\max } \right )^{2} + \left ( p_{\min } \right )^{2} + \left ( S \left ( t_{\min }, t_{\max } \right ) \right )^{2}}. $$

The daily precision at each wavelength is empirically derived as the mean of the absolute difference between the irradiance time series and the irradiance smoothed over five days. Then, the maximum and minimum precision is calculated as the daily precision divided by the square root of the number of days used for the irradiance average (217 days for solar-cycle variability). Due to the large number of days used for the solar-cycle period average, the precision terms are tiny contributions for the variability uncertainty. The stability estimates are summarized in Table 2, and details of these estimates are provided in the SORCE instrument companion articles (TIM: Kopp, 2021; SIM: Harder et al., 2022; SOLSTICE: Snow et al., 2022; XPS: Woods and Elliott, 2022). These solar-cycle variability uncertainties are shown in Figures 3 and 4, and they indicate that the UV variability is better known than the NUV-VIS-NIR variability.

There is the expectation that smoothing the irradiance over many months will average out the effects of the solar-rotation variability (e.g. center-to-limb variations) and also the effects of active-region evolution if several active regions are present at the same time. Notably, the dark-sunspots contribution for the TSI and many other wavelengths in the NUV-VIS-NIR ranges is expected to be less important than the bright-features (plage, facula, active network) contribution for averaging the irradiance over many months because the bright features dominate over the dark sunspots for about 80 % of the time (Woods et al., 2015). Consequently, the solar-cycle maximum values with several-month averages are expected to be larger than the cycle-minimum values for the TSI (as noted above) and for most wavelengths. The exception is for the darkening of facula emissions at some NIR wavelengths due to H^−^ opacity effects, and possibly also at some other visible wavelengths (Topka, Tarbell, and Title, 1997; Fontenla et al., 2011).

Based on the variability for the selected dates for the TSI and the Mg ii proxy, one expects to find that the Solar Cycle 24 spectral variability could be about a factor of two more than the Cycle 23 spectral variability for the specific dates chosen for this solar-cycle variability study during the SORCE mission. In particular, as listed in Table 3, the SORCE/TSI Cycle 24 variability to Cycle 23 variability ratio is 2.26, and the Mg ii proxy Cycle 24/23 variability ratio is 1.68. While this factor of about two in the Cycle 24 variability relative to Cycle 23 is seen for most UV wavelengths shown in Figure 3, it is not the case for the SIM results shown in Figure 4. We note that the maximum for Cycle 24 is lower than the true maximum for Cycle 23 for most wavelengths and solar proxies, but the dates chosen during the SORCE mission are not at the true maxima for either cycle.

**Table 3:**
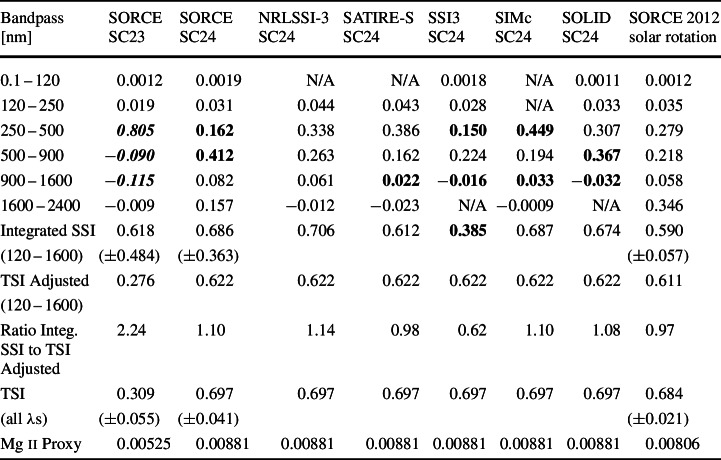
Solar-cycle irradiance variability in broad bands. Irradiance variability is calculated as maximum minus minimum and is in units of W m^−2^. The Mg ii proxy variability values are unitless. Solar Cycle 24 (SC24) values in the 120 – 1600 nm range that are more than 40 % from the SORCE 2012 solar-rotation values are indicated by **bold**. SORCE SC23 results that are not the expected factor of 2 ± 40 % lower than the SORCE SC24 values are indicated by ***bold*-*italics***.

One of our expectations from smoothing the irradiance over many months is that the spectral profile of the solar-cycle variability could be similar when selecting different dates within the same solar cycle and also for different solar cycles. However, the solar-cycle variability from the SORCE data shows noticeably different spectral variability between Solar Cycles 23 and 24. Notably, the Solar Cycle 23 variability is larger than Cycle 24 variability in the 250 – 420 nm range by almost a factor of eight, and that there are many more wavelengths with out-of-phase (negative) variability for SIM’s Solar Cycle 23 variability than for Cycle 24. These differences are quantified by the listing of the variability in broad bands in Table 3. This listing highlights that these differences from expectation are primarily for the SORCE/SIM data. Interestingly, the integrated SSI variability is very similar to the TIM TSI variability for both cycles, which partially supports the notion that SSI solar-cycle variability might be different during different solar cycles.

**Figure 5:**
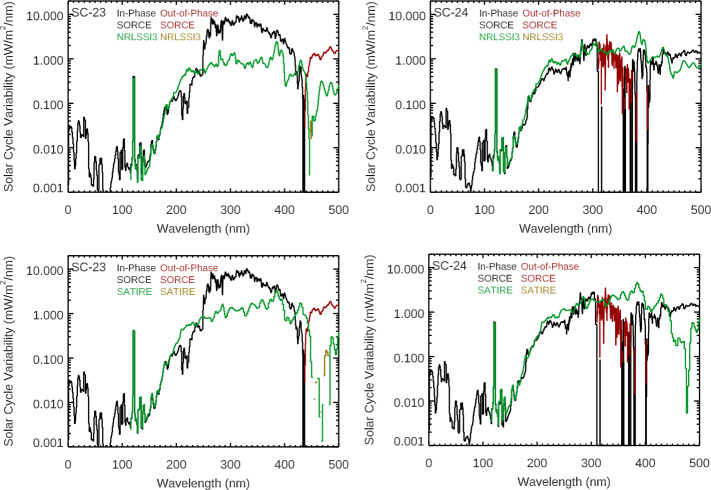
The Solar Cycles 23 (*left*) and 24 (*right*) variability results from SORCE EUV-UV-NUV ranges are compared to the NRLSSI-3 (*top*) and SATIRE-S (*bottom*) model predictions. The in-phase (positive) variability and out-of-phase (negative) variability are shown for the SORCE data and the models. There are significant differences for which wavelengths indicate out-of-phase variability for the different solar cycles for the SORCE/SIM data, whereas the models are more consistent for Solar Cycles 23 and 24. There is good agreement for the models of the solar-cycle variability for SOLSTICE (< 250 nm), but there are large differences for SIM comparison (> 250 nm).

**Figure 6:**
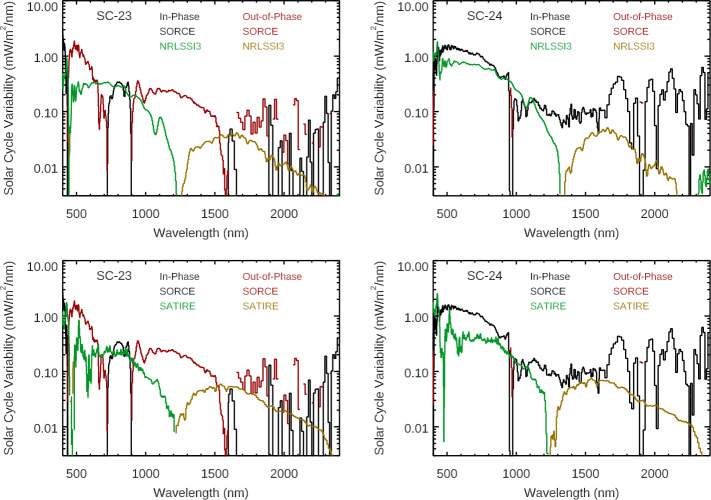
The Solar Cycles 23 (*left*) and 24 (*right*) variability results from SORCE for the VIS-NIR ranges are compared to the NRLSSI-3 (*top*) and SATIRE-S (*bottom*) model predictions. The in-phase (positive) variability and out-of-phase (negative) variability are shown for the SORCE data and the models. There are significant differences for which wavelengths indicate out-of-phase variability for the different solar cycles for the SORCE/SIM data, whereas the models are more consistent for Solar Cycles 23 and 24. There are large differences for these comparisons for most wavelengths in the VIS-NIR ranges.

Comparisons of these SORCE solar-cycle results to the NRLSSI-3 (Coddington et al., 2016; Lean et al., 2020) and SATIRE-S (Ball et al., 2014) models (see Figures 5 and 6) suggest instead that solar-cycle spectral variability might be similar for Cycles 23 and 24 and thus could indicate a concern with instrumental effects. Both models compare well for the SOLSTICE results at wavelengths less than 250 nm for both solar cycles, but neither model compares as well for the SIM results. These two models disagree the most with SIM results for Solar Cycle 23, and there are some similarities of the models to the SORCE/SIM results for Cycle 24. Both models have larger out-of-phase (negative) variability in the NIR than the SORCE results for Cycle 24. The NRLSSI-3 and SATIRE-S model predictions are more consistent with each other than they are to the SORCE solar-cycle variability. For example, both models indicate a transition from in-phase (positive) to out-of-phase (negative) variability near 1300 nm.

There have been concerns with degradation trends in the early part of the SORCE mission that could bias the Solar Cycle 23 results (e.g. Ermolli et al., 2013). The final, archived SORCE data products include all of our best estimates to date for instrumental effects (trending of dark signal and degradation and other corrections, such as from temperature changes). These corrections each have their own uncertainties (e.g. see the companion SIM algorithm article by Harder et al., 2022), and the instrument uncertainties are included as part of the SORCE data products. These uncertainties are similar in magnitude to the solar-cycle variability levels at many of the SIM wavelengths and also for the 210 – 250 nm range for the SOLSTICE data. It has been challenging to identify additional improvements for the SIM degradation trends using the flight data of the two SIM channels.

There have been a few analyses of the SORCE/SIM data that have examined and slightly adjusted the trends to make more consistent agreement of variability for Cycles 23 and 24. Woods et al. (2018) and Mauceri et al. (2020a) studied the SORCE/SIM trends and suggest slightly revised trends for the SIM data based on comparing trends to TSI and solar proxies, respectively. Their revised SSI trends are basically within the SIM uncertainties, so neither one is considered any more accurate than the official SORCE/SIM data product. The Mauceri et al. (2020a) SSI product is called the SIM constrained Version 2, or SIMc. The Woods et al. (2018) trending results for SIM are also used to make a new SSI composite called SSI3 (Woods and DeLand, 2021), and the solar-cycle variability from this SSI3 composite agrees reasonably well with the Haberreiter et al. (2017) SOLID composite that uses a different approach of using only SORCE/SIM data during Solar Cycle 24.

**Figure 7:**
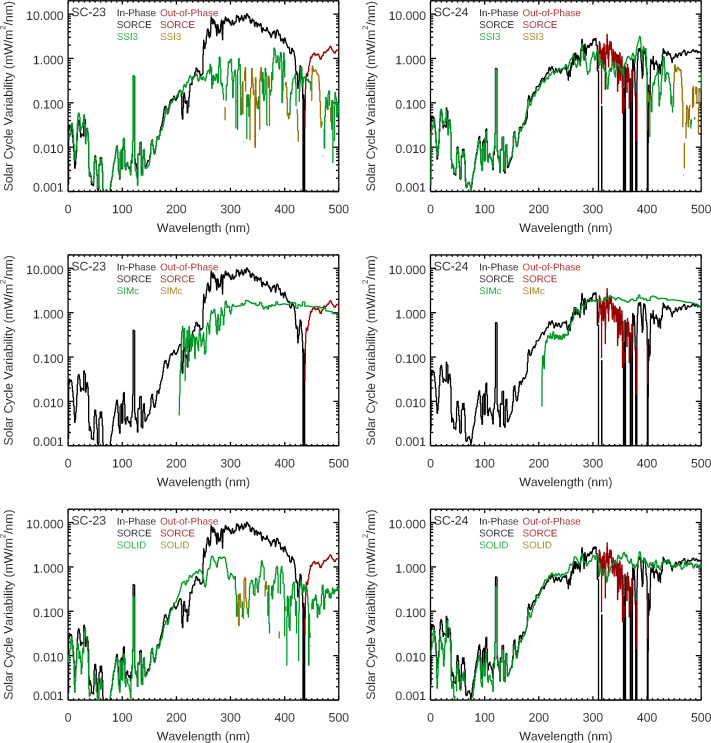
The Solar Cycles 23 (*left*) and 24 (*right*) variability results for SORCE EUV-UV-NUV ranges are compared to the SSI3 (*top*), SIMc (*middle*), and SOLID (*bottom*) composites. The inphase (*positive*) variability and out-of-phase (negative) variability are shown for the SORCE data and the composites. The composites’ spectral shapes agree better with the SORCE variability for Solar Cycle 24 for the EUV-UV-NUV ranges.

**Figure 8:**
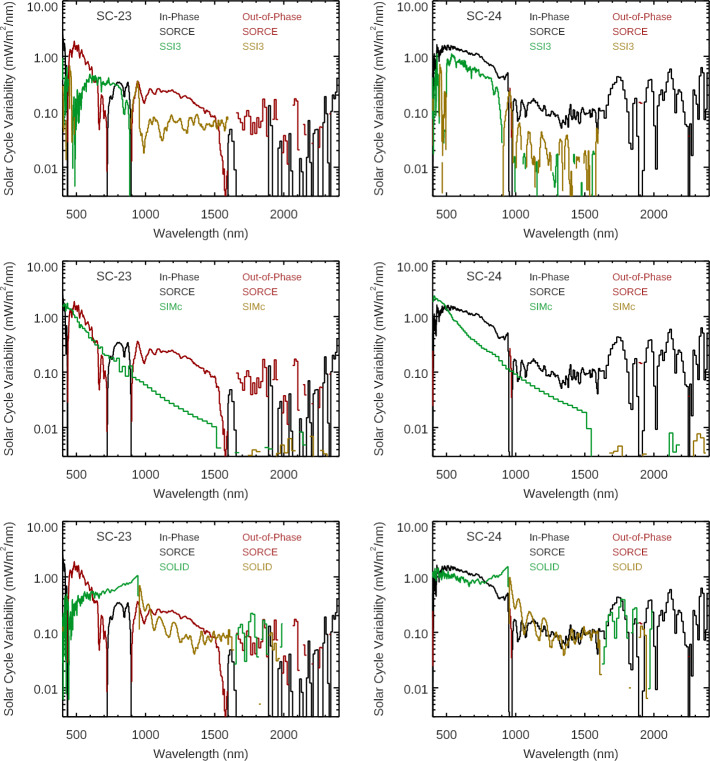
The Solar Cycles 23 (*left*) and 24 (*right*) variability results for SORCE VIS-NIR ranges are compared to the SSI3 (*top*), SIMc (*middle*), and SOLID (*bottom*) composites. The in-phase (positive) variability and out-of-phase (negative) variability are shown for the SORCE data and the composites. The composites are more consistent for which wavelengths indicate out-of-phase variability for the different solar cycles. We note that the composites’ spectral shapes agree better with the SORCE variability for Solar Cycle 24 and for wavelengths less than 900 nm.

Comparison of the solar-cycle variability for SIMc, SSI3, and SOLID composites are shown in Figures 7 and 8. All three of these composites differ more for the Solar Cycle 23 comparison than for the Cycle 24 comparison. Like the model comparison, the composites have a similar quality of good agreement to SOLSTICE ($\lambda $ < 250 nm) but have more differences from the SIM results. The spectral profile in the 500 – 900 nm range is more similar to the SSI3 and SOLID composites than to the SIMc product.

The solar-cycle results are also compared in broad bands as listed in Table 3. Because the SORCE Solar Cycle 23 results disagree more so with models and composites than for Cycle 24, this broadband listing focuses on comparing Solar Cycle 24 results. The one comparison in broad bands in Table 3 for Solar Cycle 23 is for the SORCE result, which only shows consistency for the ultraviolet range shorter than 250 nm. For the Solar Cycle 24 comparison, the NRLSSI3 model predictions agree better with SORCE than the SATIRE model, which has more NUV variability and a smaller amount of NIR variability. For the composite Cycle 24 comparisons, the SIMc has larger differences than SSI3 or SOLID. The SIMc has larger 250 – 500 nm variability than SORCE, SSI3, and SOLID, and SIMc has smaller 500 – 900 nm variability than the others. The SSI3 has smaller 250 – 500 nm variability than the others. However, this difference for SSI3 is not a SIM issue because the OMI solar data were used for the SSI3 composite for the 250 – 500 nm range.

A comparison of the integrated SSI to the TSI is also shown in Table 3. The SORCE/TIM TSI solar-cycle variability (all wavelengths) is listed in Table 3, and an adjusted TSI value for limitation to the 120 – 1600 nm range is also included for direct comparison to the integrated SSI solar-cycle variability of the same wavelength range. This TSI adjustment is calculated as the TSI variability value minus the SSI far-infrared component [W m^−2^] times the TSI relative variability (percent change). The SSI far-infrared component used for this calculation is the SORCE/TIM TSI minus the integrated SSI from SORCE, both averaged for the 2008 – 2009 minimum. That is, our assumption is that the SSI part of the TSI that is not measured by SORCE varies the same as the TSI. This seems like a reasonable assumption, especially considering that it is a small (≈ 10 %) adjustment to the TSI variability value. The SATIRE-S, SIMc, and SOLID estimates for the integrated SSI variability for Solar Cycle 24 agree best to within 10 % of the adjusted TSI value of 0.622 W m^−2^. The SORCE measurement and NRLSSI-3 also agree well to within 20 % of the adjusted TSI value. The SSI3 estimate shows the worst agreement, being 36 % lower than the adjusted TSI value; this disagreement is almost entirely due to using OMI data in the SSI3 composite for the 250 – 500 nm range. We do caution that while agreement of the integrated SSI to the adjusted TSI is important, it does not really validate the spectral variation of the SSI variability.

While this is not an exhaustive look at all possible dates during the SORCE mission for this solar-cycle variability study, the dates were carefully selected to avoid periods of instrument gaps or anomalies of the spacecraft/instruments. Furthermore, the averaging over 217 days improves the measurement precision, and the averaging over ≈ 8 solar rotations reduces the effects of center-to-limb variation and solar-activity evolution. Therefore, we expect the cases presented are the optimal results for comparing variability during Solar Cycles 23 and 24 for the SORCE data set. Our expectations are that the NUV-VIS-NIR range could vary approximately as the TSI (factor of 2.3 lower for Cycle 23 than Cycle 24 as listed in Table 3) and that the rest of the UV ranges could vary approximately as the Mg ii proxy variability (factor of 1.7 lower for Cycle 23 than Cycle 24 as listed in Table 3). As presented in Table 3, the SORCE variabilities in the EUV and UV bands of 0.1 – 120 nm and 120 – 250 nm do indeed have the expected ratio of 1.7 for Cycle 24 to Cycle 23. However, none of the other bands, which are from SIM data, have the expected ratio similar to TSI solar-cycle variability.

We find that the SSI models and composites have better agreement with SORCE Cycle 24 results than for SORCE Cycle 23 results. Because these models and composites are partially based on SORCE measurements, these comparisons do not actually verify the SORCE variability results. Instead, other independent measurements of the SSI solar-cycle variability are required and will be forthcoming soon with the TSIS-1/SIM observations once the Solar Cycle 25 activity increases. In the meantime, we have identified a special period of solar rotations during the SORCE mission that helps to identify similarities in SSI variability between measurements and models that could also relate to solar-cycle variability; this analysis is discussed in the next section.

## Irradiance Variability for Solar-Rotation Timescales

The approximate 27-day rotation of the Sun is another dominant variability component in the irradiance time series. It is modulated by the number and evolution of active regions as well as their distribution in longitude and latitude. There are also times when active regions are distributed such that 13-day variability is observed in the irradiance time series. The solar-rotational variability during the SORCE mission is discussed first, and then a special case for solar rotations in 2012 is discussed to provide some validation of the SORCE solar-cycle variability results.

### Comparison of TSI and H i Lyman-$\alpha $ Variability

The emergence of a new active region and its decay into an active network and a later quiet network affect the irradiance for about seven solar rotations (Preminger and Walton, 2006). A new active region will appear as a dark sunspot surrounded by bright faculae for NUV-VIS-NIR wavelengths but will only appear as bright regions for the UV wavelengths. The sunspot decays quickly, so the dark-sunspot effect for the TSI and NUV-VIS-NIR ranges is usually only present for its first solar rotation. Then, the bright faculae from an active region dominate for the other solar rotations, being about 80 % of the time in the TSI and SSI time series (Woods et al., 2015; Dudok de Wit et al., 2018).

**Figure 9:**
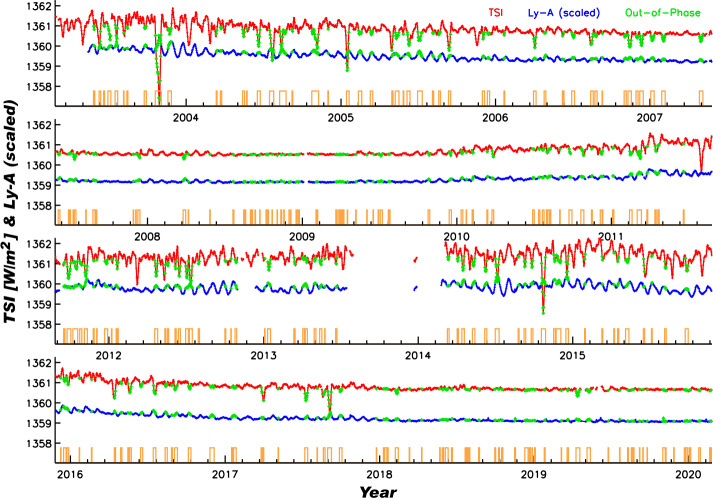
Comparisons of SORCE/TIM TSI (*red*) to the scaled SORCE/SOLSTICE Lyman-$\alpha $ (121.5 ± 0.5 nm) (*blue*) time series show in-phase variability for most days during the SORCE mission and out-of-phase behavior for the other days (*green*). The corresponding times for out-of-phase behavior are indicated in *orange* and occur 14 % of the time over the SORCE mission. This is consistent with the results of Preminger and Walton (2006) and Dudok de Wit et al. (2018), who found that the impulse response of the TSI to an emerging sunspot was negative for the first solar rotation and positive for the subsequent six, giving a similar 1/7 (14 %) ratio in time of darkening versus brightening in the TSI due to sunspots, whereas the H i Lyman-$\alpha $ emission only has positive responses during active-region evolution.

This effect for active-region evolution is clear in comparing the TSI and the H i Lyman-$\alpha $ variability as shown in Figure 9. This figure compares the SORCE/TIM TSI (red) to the scaled SORCE/SOLSTICE Lyman-$\alpha $ (121.5 ± 0.5 nm) (blue) time series. These two are generally in-phase on both solar-cycle and -rotational timescales because both respond positively to the presence of bright faculae. The TSI additionally responds to sunspots, which cause short-term decreases in brightness. However, sunspots do not decrease the Lyman-$\alpha $ irradiance, and so often they manifest short-term out-of-phase behavior between the two irradiance time series. When the TSI is lower than its average value (using a seven-rotation Gaussian-like smoothing) and when the Lyman-$\alpha $ is higher than its similar average, this condition indicates times of out-of-phase response, as shown in green. The corresponding times are indicated in orange and occur 14 % of the time over the SORCE mission. This is consistent with the results of Preminger and Walton (2006) and Dudok de Wit et al. (2018), who found that the impulse response of the TSI to an emerging sunspot was negative for the first solar rotation and positive for the subsequent six, giving a similar 1/7 (14 %) ratio in time of darkening versus brightening in the TSI due to sunspots. While the dark-sunspot effect dominates for this type of analysis, center-to-limb effects can play a role too for the phasing differences of Lyman-$\alpha $ and TSI during solar rotations.

### Spectral Variability for All Solar Rotations

**Figure 10:**
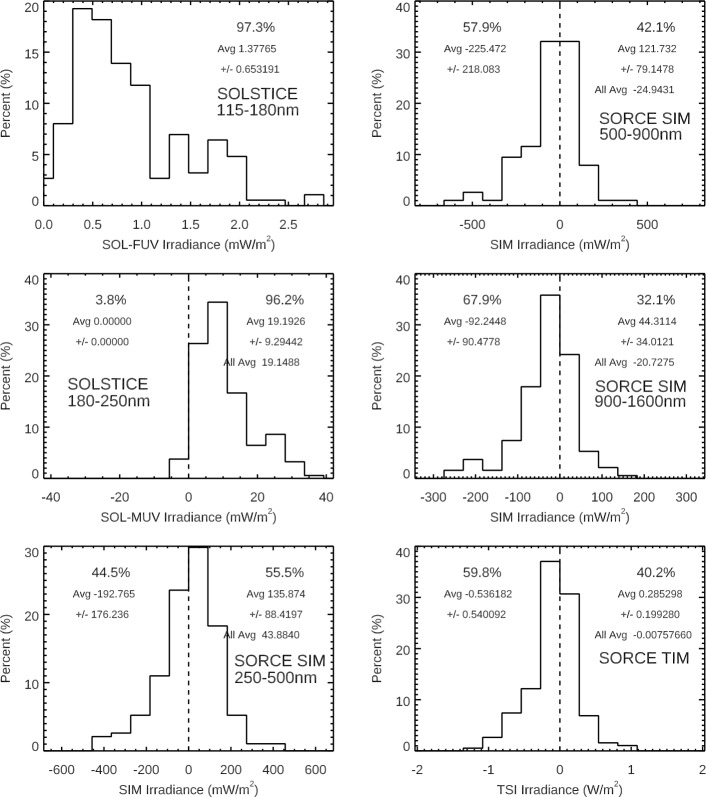
There are 250 moderate to large activity solar rotations during the SORCE mission as identified using the SORCE/SOLSTICE H i Lyman-$\alpha $ time series. The solar-rotation variability is calculated as the five-day average at each peak minus the five-day average at each valley within ±14 days of the peak, and then the distribution of those variability results is plotted for broad SSI bands and for the TSI (*bottom-right*). The solar FUV (115 – 180 nm) band behaves very much like the Lyman-$\alpha $ emission with only a positive (in-phase) distribution. Then, there is a transition to a more and more negative (out-of-phase) distribution going up in wavelength. The SSI 500 – 900 nm band is the band that is most similar to the TSI result.

Another study about irradiance variability on solar-rotation timescales is the examination of all observed SSI wavelengths to explore the distribution (phasing) of the SSI rotational variability relative to Lyman-$\alpha $ and TSI. For this study, we identified all of the peaks and valleys within 14 days of the peaks using the SORCE/SOLSTICE H i Lyman-$\alpha $ emission (SORCE SSI Level 3 121 – 122 nm irradiance). Only the Lyman-$\alpha $ rotational variabilities with more than 3% variation are selected for this study, thus representing solar rotations with moderate to large variability. The range of rotational variability for Lyman-$\alpha $ for this set is from 3 % to 30 % and with a mean of 12 % for 205 solar-rotation periods. Then, for each wavelength, the irradiance variability for each solar-rotation period is calculated as the five-day average at each peak minus the five-day average at each associated valley. Then, the distribution of those variability results is examined to identify positive (in-phase with Lyman-$\alpha $) and negative (out-of-phase with Lyman-$\alpha $) variability for solar-rotation timescales. The presented averages are weighted with the amount of the Lyman-$\alpha $ variability so that the more active solar rotations have more importance. We note that the average without this weighting is typically about 15 % smaller than the weighted average because there are many more solar rotations with smaller variability. Figure 10 shows examples of this rotational timescale variability distribution for broad SSI bands and also for the TSI.

**Figure 11:**
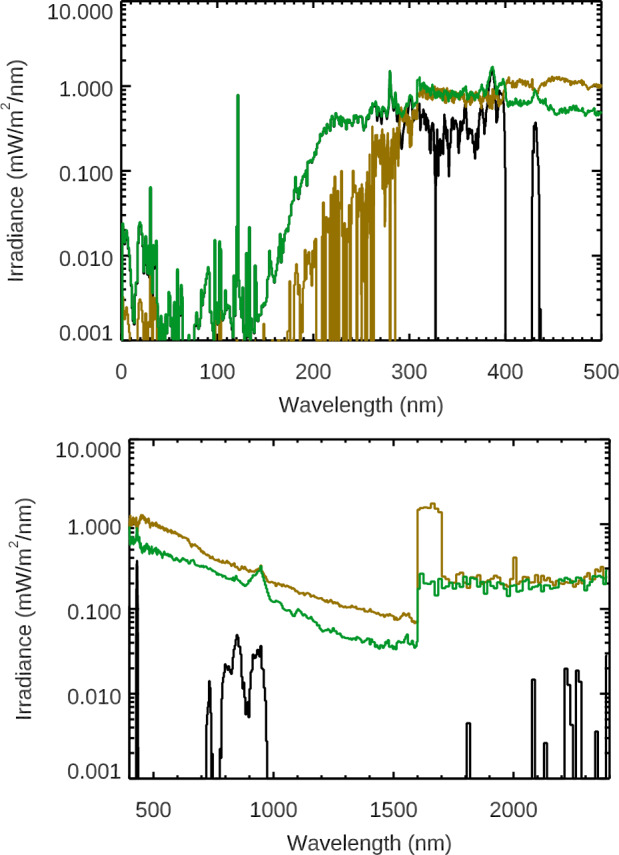
These variability results are for 250 solar rotations during the SORCE mission. The average of all of these solar rotations is in *black*. For each wavelength, the average of the solar rotations with positive variability, being in-phase with Lyman-$\alpha $, is in *green*, and the negative out-of-phase variability average in *gold* is inverted for this logarithmic scale. There are distinct changes in behavior at 290 nm, 400 nm, and 1600 nm, as discussed in the text.

The SOLSTICE FUV (115 – 180 nm) and MUV (180 – 250 nm) solar emissions behave very much like the Lyman-$\alpha $ emission with most wavelengths having only a positive (inphase) distribution. There is a transition to a more negative (out-of-phase) distribution for wavelengths longer than 290 nm, as shown in Figure 11. There is about an equal distribution of positive and negative rotational timescale variability for 290 – 400 nm and 1700 – 2400 nm, and there is more negative rotational variability than positive for 400 – 1700 nm. The variability distribution in the 1600 – 2400 nm range appears to be related to the measurement precision being comparable to the amount of rotational timescale variability; the results are more statistically significant for all other wavelengths shorter than 1600 nm.

One interesting result is the similarity of the rotational timescale variability distribution of the SSI 500 – 900 nm band to the TSI result, as shown in Figure 11. This similarity to the TSI is partially related to the irradiance peaking in the visible band. In both cases, there is about an equal (50/50) distribution of positive and negative rotational timescale variability. This 40/60 ratio of in-phase to out-of-phase for the TSI solar rotations is very different from the 86/14 ratio presented earlier for all days during the SORCE mission (Figure 9) because this distribution in Figure 10 is limited to the moderate and large activity solar rotations. As noted in Figure 10, the character of the rotational timescale variability is more positive for the 250 – 500 nm band, especially so for individual wavelengths below 290 nm. Also, the rotational timescale variability is more negative for the 900 – 1600 nm band, both for the broad band and individual wavelengths. As discussed earlier, the differences for being in-phase and out-of-phase relative to Lyman-$\alpha $ variability are largely related to the dark-sunspot effects for the NUV-VIS-NIR ranges and also partly due to differences in the center-to-limb effects for different emissions.

### Variability for Special Solar-Rotation Period in 2012

**Figure 12:**
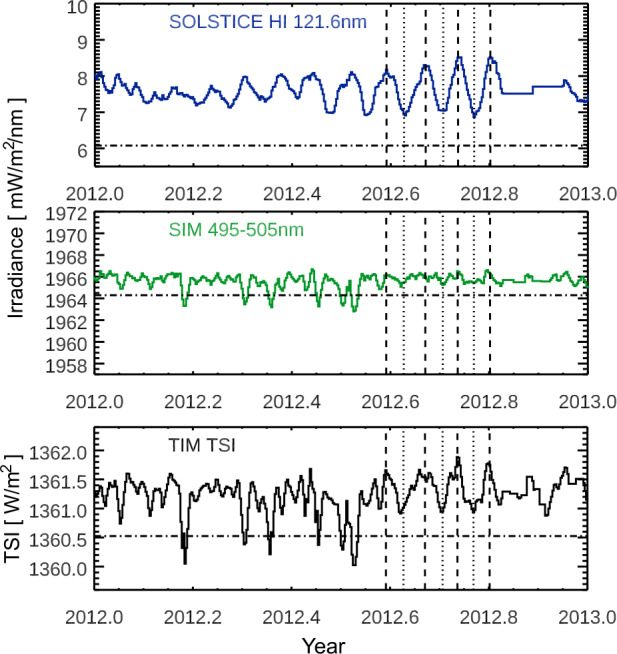
The solar-rotation timescale variability is determined from the four solar rotations during the latter part of 2012, as indicated with *dashed lines* for the rotation peaks (2012 Days 217, 246, 270, and 294) and *dotted lines* for the rotation valleys (2012 Days 230, 259, and 282). The *dot–dashed line* is the irradiance during the 2008 – 2009 minimum. These time series include five-day smoothing of the SORCE data.

We also examined the solar-rotation timescale variations over the full SORCE mission to identify a period when the TSI and UV variations are in-phase over multiple, consecutive solar rotations. The motivation for selecting this period is that there could be spectral consistency between the solar-rotation timescale variability and the solar-cycle variability if these solar rotations do not have dark-sunspot effects. That is, this special in-phase period has the potential to partially validate the SORCE solar-cycle variability results. The selection of dates for this special solar-rotation study is a period in 2012 of four solar rotations that starts one solar rotation after new active regions have emerged. This 2012 period is from 4 August (DOY 217) to 20 October (DOY 294), as shown in Figure 12. This 2012 period is also interesting because its solar-rotation variability magnitude is one of the largest during the SORCE mission.

**Figure 13:**
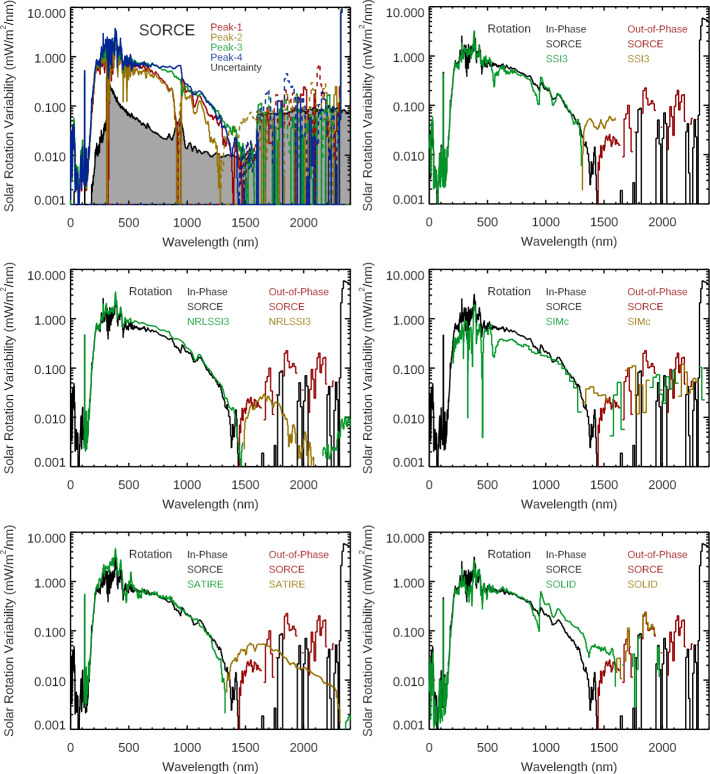
The *top-left panel* shows the SORCE solar-rotation variability for the four rotation peaks (2012 Days 217, 246, 270, and 294), along with the variability uncertainty (*black line and grey-shaded region*). The *middle-left panel* shows the comparison of the average of the SORCE rotation variabilities to the NRLSSI-3 model predictions for those solar rotations. The *bottom-left panel* shows the comparison of the average of the SORCE rotation variabilities to the SATIRE-S model predictions. The *right panels* show the comparison for the SSI3 (*top*), SIMc (*middle*), and SOLID (*bottom*) composites. The model predictions and composites agree much better with the SORCE rotational variability than for the solar-cycle variability comparisons.

The TSI and NUV-VIS-NIR wavelengths are in-phase with the UV emissions for those four solar rotations, as illustrated in Figure 12. The solar rotation prior to this period shows the effect of the dark sunspots for the TSI and SSI at 500 nm. To obtain the solar-rotation variability, three-day averages are taken at rotation peaks and valleys, and then the four peak values are averaged together for the rotation maximum and the three valley values are averaged for the rotation minimum. This solar-rotation variability with SORCE data is shown in Figure 13 along with a comparison of the solar-rotation variability from the two models and three composites calculated in the same way. The SORCE 2012 solar-rotation variabilities in broad bands are also listed in Table 3. Interestingly, the TSI variation for this solar-rotation period has a very similar magnitude as the TSI variation for the Solar Cycle 24 study.

The solar-rotation variability for each of the four rotation peaks is shown in the top panel of Figure 13. The average of the three rotation valleys is used for this plot showing variability for each peak. The average of the four solar-rotation peaks is used for the other plots in Figure 13. While we do not expect the four rotational peaks to have identical variability, we were expecting them to have consistencies in spectral profile. There are two regions where there is not consistency. One is near 900 nm, which is where the SIM visible photodiode has thermal corrections that are challenging (i.e. large temperature coefficient of radiant sensitivity). The other is the 1600 – 2400 nm region where the SIM ESR is used and has less precision than the SIM photodiodes, partly due to its technology and partly due to the ESR fixed-wavelength observations being more sensitive to wavelength shifts dependent on temperature changes. Averaging the four rotation variabilities improves the 900-nm region, but the 1600 – 2400 nm region still has rapidly changing spectral changes (positive and negative) that may not be realistic.

The solar-rotation timescale variability uncertainty is primarily from the measurement precision because the stability estimate is over such a short period of time (relative to solar-cycle variability). This variability uncertainty is calculated in the same way as described above for the solar-cycle timescale variability uncertainty. These uncertainties for the SORCE solar-rotation results are shown as the gray-shaded region in the top-left panel of Figure 13.

Comparison of the SORCE 2012 solar-rotation timescale variability to the NRLSSI-3 and SATIRE-S model shows good agreement up to 1600 nm. The NRLSSI-3 model predictions are more consistent with SORCE, including the transition from in-phase to out-of-phase near 1400 nm. The SATIRE-S model has this transition near 1300 nm, has a larger out-of-phase amplitude in the NIR than SORCE or NRLSSI-3, and has larger variability in the 200 – 400 nm range than SORCE or NRLSSI-3. There is not agreement in the 1600 – 2400 nm range for either model, and this is thought to be the limitation of the SIM ESR measurement precision. We note that the measurement precision has significantly improved for later-generation SIM instruments aboard TSIS-1 and CSIM.

Comparisons for solar-rotation timescale variabilities are expected to be more precise for any instrument because the amount of instrument degradation and other trends are much smaller corrections for shorter periods of time. There are much improved spectral consistencies of the variabilities from the four different rotation peaks than for the solar-cycle results. As we chose not to include a solar rotation with dark-sunspot effects, we anticipate that there should be spectral consistency between the solar-rotation timescale variability and the solar-cycle variability. That is the case for the SORCE data from XPS and SOLSTICE, but it is not the case for SIM. For SIM, there is reasonable agreement within instrument uncertainties for its Solar Cycle 24 variability, especially so between 250 nm and 1300 nm. The large differences for SIM’s Solar Cycle 23 variability suggests that degradation trends still need improvements for the earlier part of the SORCE mission.

## Comparison of Solar Minima

**Figure 14:**
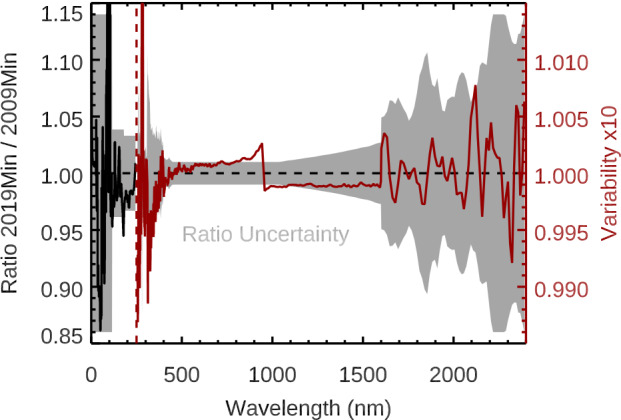
The differences between the 2019 – 2020 minimum and 2008 – 2009 minimum for the SORCE SSI data do not indicate a significant long-term change because those differences are mostly within the SORCE one-$\sigma $ uncertainties (*gray region*).

Potential SSI changes between minima are likely to be small relative to the minimum-to-maximum solar-cycle amplitude. Any such changes between minima could be indications of secular changes or trends. There were some indications that the 2008 – 2009 solar-minimum irradiance was lower than the 1996 minimum irradiance in the EUV by 10 ± 6 % (Solomon et al., 2011) and for the TSI by 140 ± 106 ppm (Fröhlich, 2009). Those potential changes are small compared to the typical solar-cycle variability of a factor of 2 – 3 for the EUV range and 700 – 1000 ppm for the TSI. Comparisons of minima with the same instruments are far more accurate than comparisons of minima from different instruments, which usually have offsets in their irradiance levels unrelated to long-term solar trends. The SORCE 17-year mission provides a special opportunity to compare the 2008 – 2009 minimum to the 2019 – 2020 minimum with the same set of instruments. The comparison of these minima with the 217-day smoothing is shown in Figure 14. Those SSI minima differences do not indicate a significant long-term change because the differences are mostly within the SORCE one-$\sigma $ uncertainties. The integrated SSI irradiance values are 1321.62 W m^−2^ for the 2008 – 2009 minimum and 1321.57 W m^−2^ for the 2019 – 2020 minimum. The one-$\sigma $ uncertainty for the integrated SSI is estimated to be 2.8 W m^−2^, or 2100 ppm. The change of the integrated SSI with the SORCE data over this 11-year period is 0.05 W m^−2^, or a 37 ppm change. This small change is insignificant relative to the integrated SSI uncertainty of 2100 ppm.

The TIM TSI values, also with 217-day smoothing, are 1360.523 W m^−2^ for the 2008 – 2009 minimum and 1360.667 W m^−2^ for the 2019 – 2020 minimum. This change is a TSI 11-year increase of 0.144 W m^−2^, or a 106 ppm change. We note that the TSI change suggests an increase, whereas the integrated SSI change suggests a decrease. The TIM one-$\sigma $ uncertainty for this TSI difference is 110 ppm, so this TSI difference is also considered to not be a significant result. Our conclusion is that the 2019 – 2020 minimum is very similar to the 2008 – 2009 minimum to within the SORCE measurement one-$\sigma $ uncertainty.

## Conclusions

The SORCE solar-irradiance observations span a 17-year period covering the decline of Solar Cycle 23 and covering the full period of Solar Cycle 24. This long mission provides important data for Sun–climate studies of the TSI and SSI in the wavelength ranges of 0.1 – 27 nm and 115 – 2400 nm. Companion articles about the SORCE instruments and their final science data-processing algorithms provide additional details about individual instrument measurements over the course of the SORCE mission. The focus here is on the solar-cycle variability during the SORCE mission. As a validation check for the solar-cycle study, a special period in 2012 is examined for solar-rotation timescale periods that might reflect spectral variability similar to solar-cycle timescale variations. This period includes four solar rotations where TSI and UV irradiances are in-phase with each other (that is, without any strong dark-sunspot effects). The key conclusions from this study are the following:

- The SORCE solar-cycle variability results show spectral consistency between Cycles 23 and 24 for wavelengths less than 250 nm from SORCE/XPS and SORCE/SOLSTICE.
- The differences for the SIM solar-cycle results include higher UV variability in the 250 – 420 nm range for Cycle 23 than for Cycle 24 and more out-of-phase (negative) variability in the visible and NIR for Cycle 23 than for Cycle 24. Comparisons with a special solar-rotation period in 2012 suggest that the SIM results for Solar Cycle 24 might be more accurate than the Cycle 23 results. We note that the SORCE/SIM solar-cycle variability results are about one-$\sigma $ results, and the solar-rotation variability results are about ten-$\sigma $ results.
- The SORCE/SIM variability results for the 1600 – 2400 nm range are more noisy. They are not consistent across the two solar cycles or for the solar-rotation periods studied, nor are they consistent with the models. The SORCE/SIM 1600 – 2400 nm results may not reflect true solar-cycle variability.
- The spectral profile for the SORCE 2012 solar-rotation study partially validates the SORCE variability results for Solar Cycle 24, but not for Cycle 23 variability for wavelengths more than 250 nm from the SIM.
- The NRLSSI-3 and SATIRE-S model predictions for solar-cycle variability agree well with the SORCE results for wavelengths less than 250 nm and have reasonable agreement with the SORCE/SIM results up to 1300 nm for Solar Cycle 24 but not for Cycle 23.
- The NRLSSI-3 and SATIRE-S model predictions for the chosen 2012 solar-rotation period agree very well with the SORCE results up to 1600 nm. The NRLSSI-3 model has slightly better agreement than the SATIRE-S model for the comparisons to the SORCE solar-rotation variability.
- The SSI3, SIMc, and SOLID composites have solar-cycle variability results that agree better with the SORCE Solar Cycle 24 results than with the Cycle 23 results. The SSI3 and SOLID composites have spectral profiles for the Solar Cycle 24 and solar-rotation timescale variabilities that behave more like the SORCE results than for the SIMc product.
- The SORCE observations of the 2008 – 2009 cycle minimum and the 2019 – 2020 cycle minimum indicate that these two minima have very similar levels, being within their measurement one-$\sigma $ uncertainties, so we conclude that there is minimal, if any, long-term (secular) trend of the solar irradiance over the SORCE mission.

The SORCE mission has been an important NASA Earth Science mission that helped continue the 43-year-long TSI and SSI UV climate-data records and initiated the SSI NUV-VIS-NIR climate record. There are still unresolved solar-cycle variability results, mostly for Cycle 23, from the first-generation SIM instrument onboard SORCE. We note that the integrated SSI has similar variability as the TSI variability and that the special 2012 solar-rotation variability has similar spectral characteristics as the solar-cycle variability up to 1600 nm, but neither comparison fully validates the SORCE solar-cycle variability results. We anticipate that improved solar-cycle variability results will be forthcoming with the more accurate observations from the second- and third-generation SIM instruments onboard TSIS-1 and CSIM, respectively. The end of the SORCE mission was a planned passivation of the spacecraft following a successful two-year overlap with the TSIS-1 mission, which continues the TSI and SSI climate records. The TSIS-1 SSI observations span 200 to 2700 nm, extending further into the IR than SORCE but leaving a spectral gap below 200 nm. The solar activity during Solar Cycle 25 finally began its ramp up in late 2021, so we anticipate that the TSIS-1/SIM and CSIM observations in early 2022 will significantly improve upon the solar-cycle variability understanding for the 300 – 2700 nm range.

## Data Availability

The SORCE public data products are described on the SORCE website at lasp.colorado.edu/sorce/data/. Data files, and the software to read them, are available for direct download from this web site. SORCE data products are delivered and archived at the Goddard Earth Science (GES) Data and Information Services Center (DISC) at disc.gsfc.nasa.gov/datasets?page=1&keywords=sorce. The SORCE archived documents are also on the DISC at disc.gsfc.nasa.gov/information/documents?keywords=SORCE&title=SORCE Mission Preservation Documents. In addition, the LISIRD website at lasp.colorado.edu/lisird/ provides interactive access to the SORCE solar-irradiance data, where they coexist with related solar-irradiance data products from other missions.
